# Hypoglycemic effect of *Taraxacum officinale* root extract and its synergism with *Radix Astragali* extract

**DOI:** 10.1002/fsn3.2176

**Published:** 2021-02-26

**Authors:** Jingwen Li, Jiayuan Luo, Yangyang Chai, Yang Guo, Yang Tianzhi, Yihong Bao

**Affiliations:** ^1^ School of Forestry Northeast Forestry University Harbin China; ^2^ Key Laboratory of Forest Food Resources Utilization of Heilongjiang Province Harbin China; ^3^ Department of Agricultural, Food and Nutritional Science University of Alberta Edmonton AB Canada

**Keywords:** combination, hypoglycemic effect, *Radix Astragali*, synergism, *Taraxacum officinale* root

## Abstract

*Taraxacum officinale* (dandelion) and *Radix Astragali* are traditional medicinal and edible plants with high nutritional value. In this study, the synergistic hypoglycemic effect of DRE and *Radix Astragali* extract (RAE) was evaluated. Our results showed that water extract of dandelion (DRE‐w), mainly containing polysaccharides (63.92 ± 1.82 mg/g), total flavonoid (2.57 ± 0.06 mg/g), total phenolic compounds (8.93 ± 0.34 mg/g), and saponins (0.54 ± 0.05 mg/g), exhibited significantly inhibitory effect on α‐glucosidase and α‐amylase. DRE‐w and RAE had synergistic hypoglycemic effect; we found that DRE‐w and its combination with RAE could relieve the state of insulin resistance in IR‐HepG2 cells. The combination could more significantly increase the glucose consumption and intracellular glycogen content, and improve the activity of hexokinase and pyruvate kinase in IR‐HepG2 cells. In summary, DRE and its combination with RAE can be developed as the drugs or functional foods for diabetes prevention and treatment.

## Graphical abstract

In this study, the effect of dandelion root extracts (DREs) on the hypoglycemic effect and analysis of active compounds were investigated. We found that DRE and RAE had synergistic hypoglycemic effect. We believe that the results of this research are significant to the development of functional foods and healthcare drugs with hypoglycemic effects.

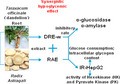

## 1 INTRODUCTION

Diabetes mellitus (DM) is a chronic noninfectious disease which is caused by abnormal secretion or abnormal function of insulin. According to the statistics of the International Diabetes Federation (IDF) (https://diabetesatlas.org/en/), about 463 million people are deeply troubled by DM at present. DM has become a kind of worldwide metabolic disease. Clinically, DM is mainly divided into three types: type 1 diabetes mellitus (T1DM), type 2 diabetes mellitus (T2DM), and gestational diabetes mellitus (GDM) (Ogurtsova et al., 2017). Ninety percent of diabetics are suffering from T2DM worldwide (Cho et al., 2018). Hyperglycemia is a typical feature of DM (Park et al., 2020); it can cause damage to organs and tissues and further cause complications such as cardiovascular disease, kidney disease, and eye disease (Huang et al., 2018; Schuett, 2020; Thomas et al., 2019). Therefore, it is necessary to take hypoglycemic drugs in time for diabetics to control level of blood glucose, so as to decrease and control the occurrence of complications.

Drugs for treating diabetes commonly used include metformin, glibenclamide, and acarbose which can increase the utilization of glucose and reduce the output of liver glucose (Dash et al., 2018). Although traditional medicine can effectively exert therapeutic effects, they still have some side effects such as vomiting and diarrhea (Wang et al., 2017). Currently, consumers pay more and more attention to natural products due to high efficacy and low toxicity. Many bioactive compounds such as polysaccharides and saponins in plants have been proven to have hypoglycemic effects (Liang et al., 2017; Su et al., 2020; Zhao et al., 2020). Moreover, the combination of different drugs is considered more effective on treatment of diabetes, which may be due to the complementary role in the mechanism of different biological compounds (Ding et al., 2019; Jiang et al., 2020). It is worth noting that combinations of different drugs in low dose are more common, which can solve the problems of drug dependence, side effects, and tolerance of high dose to some extent (Hu et al., 2019; Sekar et al., 2019). Therefore, the application of natural products in combination can be regarded as a safer and more effective way.

Dandelion (*Taraxacum officinale*) is a herbaceous plant of the Asteraceae family. Abundant resources of dandelion can be obtained around the world (Yang & Zhang, 2020). Its flowers, leaves, and roots all have edible and medicinal value (Guo, Zhang, et al., 2019; Guo, Niu, et al., 2019). Dandelion contains amounts of polysaccharides, flavonoids, phenolic compounds, and terpenes (Diaz et al., 2018). These compounds decide that dandelion has biological functions of anticancer, anti‐inflammatory, antidiabetes, and antirheumatism (Gonzalez‐Castejon et al., 2012; Guo et al., 2019a; Guo, Niu, et al., 2019). Recent scientific researches showed that DRE could relieve the symptom of colitis in experimental mice and its abundant phenolic compounds showed excellent antioxidant property (Ding & Wen, 2018; Wirngo et al., 2016). Studies have pointed out that DRE also had a therapeutic effect on leukemia of patients (Kenny et al., 2015). However, there are limited reports on the applications of dandelion root in diabetes which need to be further studied and confirmed. *Radix Astragali* is a common natural herb. It is the most frequently used Chinese medicine for the treatment of diabetes. The main components identified in water extract of *Radix Astragali* (RAE) are isoflavonoids, polysaccharides, triterpenoid saponins, and aminobutyric acid by chemical detection, among which isoflavonoids are the marker compounds (Alzoman et al., 2019). Recent studies have showed RAE could effectively inhibit the activity of α‐glucosidase (Wang, He, et al., 2020; Wang et al., 2020). In vivo experiments, RAE could relieve the typical symptoms of T2DM rats; it participated in 14 metabolic pathways of rats and influenced 22 metabolites (Liu et al., 2017). Furthermore, RAE could control abnormal oxidative stress level in diabetic rats, thereby protecting the kidneys of rats from damage (Guo et al., 2019a, 2019b).

Accordingly, in this study, the content of potential natural composition in DRE was determined by quantitative measurement. On this basis, the inhibitory effects of DRE, RAE, and their combination on α‐amylase and α‐glucosidase were studied. The interaction between DRE and RAE was also determined. Additionally, the HepG2 cells were used to establish an insulin resistance model to further evaluate their hypoglycemic activity through the impact on glucose metabolism, which might provide a theoretical basis for the development of related hypoglycemic products.

## 2 MATERIALS AND METHODS

### 2.1 Materials and chemicals

Dandelion roots were collected from Nanzhao, Henan Province, China. *Radix Astragali* was purchased from Heilongjiang Province. Dandelion roots and *Radix Astragali* were sieved (40 mesh) to obtain fine powder and stored at −4°C until use. α‐Glucosidase, α‐amylase, and 4‐nitrophenzl‐α‐D‐ glucopyranoside (PNPG) were obtained from Shanghai Yuanye Biotechnology Co., Ltd. Other chemicals and reagents were of analytical grade and were obtained from Tianjin Guangfu Fine Chemical Research Institution. All kits were purchased from Nanjing Jiancheng Bioengineering Institute.

### 2.2 Preparation of extract

The dandelion root powder was extracted with different solvent at a ratio of 1:20 (m/V) by reflux treatment for 2 hr to obtain the dandelion root extracts of distilled water (DRE‐w), methanol (DRE‐m), ethanol (DRE‐e), n‐hexane (DRE‐n), ethyl acetate (DRE‐a), and chloroform (DRE‐c), respectively. The extract was collected and lyophilized to obtain powders and stored at −20°C until use. *Radix Astragali* extract (RAE) was prepared with distilled water as extraction solvent by above method. The yield of DRE was calculated as follows:

$$\text{yield}\;\left(\%\right)=\frac{m_{1}}{m_{2}}\times 100\text{\textbackslash{}\%}\;$$

*m*_1_: weight of extract (g), *m*_2_: weight of sample powder (g).

#### 2.2.1 Quantitative analyses of biologically active compounds in DRE

The contents of total flavonoid, saponins, polysaccharides, and total phenolic compounds in DRE were determined by standard methods.

Total flavonoid content of DRE was determined by the aluminum chloride colorimetric method (Gao et al., 2012; Sopee et al., 2019). 2 ml of DRE (5 mg/ml) and 0.3 ml AlCl_3_ solution (1 mol/L) were added in a test tube and incubated at room temperature for 6 min. Then, 2.5 ml NaCOOH (2 mol/L) was added and the volume was made up to 5 ml with ethanol. The reaction solution was incubated for 40 min. The absorbance was recorded at 460 nm. Rutin was selected as the standard; the regression equation of standard curve was obtained by this method as *Y* = 8.2514*x* + 0.0023 (*R* ^2^ = .9998).

The saponin content of DRE was determined by the coloration method (Chelladurai & Chinnachamy, 2018). 1.0 ml of DRE (5 mg/ml), 0.8 ml of perchloric acid, and 0.2 ml of vanillin solution (0.5%) were added into a test tube, mixed thoroughly, and incubated in a water bath at 60°C for 20 min. Then, the reaction solution was allowed to cool in an ice bath for 5 min, followed by the addition of 5 ml ice acetic acid. The absorbance was measured at 554 nm. Sarsasapogenin was selected as the standard; the regression equation of standard curve was obtained by this method as *Y* = 0.2027*x* − 0.2145 (*R* ^2^ = .9992).

The polysaccharide content of DRE was determined by the phenol‐sulfuric acid method (Serventi et al., 2013). 1.0 ml of DRE was mixed with 1 ml of phenol solution (5%), and 6.0 ml of concentrated sulfuric acid was added. The reaction solution was allowed to stand for 30 min at room temperature, before the absorbance was measured at 490 nm. Glucose was selected as the standard; the regression equation of standard curve was obtained by this method as *Y* = 8.617*x* + 0.0451 (*R* ^2^ = .9991).

The content of total phenolic compounds in extracts was determined by the coloration method (Lim et al., 2020; Xie et al., 2019). 1.0 ml of DRE was mixed with 1 ml of foline‐phenol solvent. After incubated for 3 min at room temperature, 3 ml of Na_2_CO_3_ (10%) was added. The absorbance was measured at 750 nm. Gallic acid (GA) was selected as the standard; the regression equation of standard curve was obtained by this method as *Y* = 0.1071*x* − 0.126 (*R* ^2^ = .9993).

### 2.3 α‐Glucosidase inhibition assay

The assessment of inhibitory activity against α‐glucosidase was based on reported methods (Lordan et al., 2013; Tao et al., 2020). α‐Glucosidase and PNPG were dissolved in phosphate buffer solution (0.1 mol/L, pH 6.8). 10 μl of the test sample solution (5mg/ml) was mixed with 40 μl of the α‐glucosidase solution (4 U/ml) and added into a 96‐well cluster plate, incubated at 37°C for 10 min. Then 50 μl of PNPG (2 mmol/L) was added to the reaction mixture. After incubated at 37°C for 1 hr, the reaction was terminated by adding 50 μl of Na_2_CO_3_ (0.1 mol/L). The absorbance was measured at 405 nm. Acarbose was selected as positive control. The IC_50_ value (the concentration of inhibitors at which the inhibitory rate of enzyme is 50%) was determined. Correlation coefficients between each active composition in extract and inhibitory rate were calculated using SPSS 22.0 software. The inhibitory rate was calculated as below:

$$\%\;\text{Inhibition}=\left(1‐\frac{A_{\text{sample}}‐A_{\text{background}}}{A_{\text{control}}}\right)\times 100\%$$

*A*_sample_: Absorbance of test sample + enzyme + PNPG. *A*_background_: Absorbance of test sample without enzyme. *A*_control_: Absorbance of 100% enzyme + PNPG without test sample.

### 2.4 α‐Amylase inhibition assay

The assessment of inhibitory activity against α‐amylase was based on reported methods (Shodehinde et al., 2017; Xu et al., 2019). α‐Amylase solution and starch solution were prepared with phosphate buffer solution (0.1 mol/L, pH 6.8). 200 μl of α‐amylase solution (1 U/ml) and 100 μl of test samples solution (5 mg/ml) were sequentially added in the test tube and incubated at 37°C for 5 min. After that, 500 μl of the starch solution (10 g/L) was added and incubated at 37°C for 5 min. The reaction was terminated by adding 500 μl of DNS and was allowed to cool in ice water after incubation in a boiling water bath for 5 min. The reaction mixture was diluted by distilled water to 25 ml. The absorbance was measured at 540 nm. Acarbose was selected as the positive control. Correlation coefficients between each active composition in extract and inhibitory rate were calculated using SPSS 22.0 software. The inhibitory rate was calculated as below:

$$\%\;\text{Inhibition}=\left(1‐\frac{A_{\text{sample}}‐A_{\text{background}}}{A_{\text{control}}}\right)\times 100\%$$

*A*_sample_: Absorbance of test sample + enzyme + PNPG. *A*_background_: Absorbance of test sample without enzyme. *A*_control_: Absorbance of 100% enzyme + PNPG without test sample.

### 2.5 Determination of combination index and interaction

For evaluating the effect of ratio of DRE and RAE in combination (5 mg/ml) on α‐glucosidase inhibition, the DRE and RAE were combined by the ratio of 3:1, 2:1, 1:1, 1:2, and 1:3, and the IC_50_ values were measured as an indicator for judgment. Then, the analysis was based on the median‐effect principle (Chou‐Talalay) (Chou & Talalay, 1984; D'Ascola et al., 2019), and the median‐effect equation was presented as *f*_a_/*f*_u_ = (*D*/*D*_m_)^m^, where *D* is the dose; *f*_a_ and *f*_u_ are the fractions of effective and ineffective part by the dose *D*; *D*_m_ is the dose that produces the median effect; and m represents the Hill‐type coefficient of the dose–response curve. From this, the median‐effect plot was generated. The mode of interaction between DRE and RAE on α‐glucosidase inhibition was assessed by Combination Index (CI), where CI > 1 indicated antagonism, CI = 1 indicated additive, and CI < 1 indicated synergism. The CI was calculated as below:

$$\text{CI}=\frac{{\left(D\right)}_{1}}{{\left(D_{x}\right)}_{1}}+\frac{{\left(D\right)}_{2}}{{\left(D_{x}\right)}_{2}}$$

(*D*)_1_ and (*D*)_2_: the concentrations of DRE and RAE in drugs combination that are able to induce 50% of inhibitory rate. (*D*_x_)_1_ and (*D*_x_)_2_: the concentrations of DRE and RAE that are able to induce 50% of inhibitory rate.

### 2.6 Induction of insulin resistance HepG2 (IR‐HepG2) model

HepG2 cells were grown in complete medium (DMEM with 10% fetal serum and 1% combined antibiotics) at 37°C in a 5% CO_2_ humidifying atmosphere. Cells at logarithmic growth phase were used for subsequent experiments (Liu et al., 2019).

Induction of IR‐HepG2 model was based on previous researches (Cao, Li, et al., 2019; Cao, Zhang, et al., 2019). HepG2 cells were seeded in a 96‐well plate at 1 × 10^5^ cells/well and cultured for 24 hr. Afterward, the cells were treated with different concentration of insulin in complete medium, respectively: 10^–9^, 10^–8^, 10^–7^, 10^–6^, and 10^–5^ mol/L for 36 hr to obtain IR‐HepG2 model. The content of glucose in the culture media was measured by a glucose kit. The concentration of insulin for modeling was selected according to the glucose consumption of cells.

### 2.7 MTT assay

HepG2 cells were seeded in a 96‐well plate at 1 × 10^5^ cells/well and cultured for 24 hr. After that, the culture media of each well was replaced with fresh culture media containing different concentrations of DRE, RAE, and their combination (CB) (respectively: 1, 2, 3, 4, 5 mg/L). After incubated for 36 hr at 37°C, the media of each well was replaced with 90 μl of fresh medium and 10 μl of 3‐(4,5‐dimethylthiazol‐2‐yl)‐2,5‐diphenyltetrazolium bromide (MTT) solution, followed by incubation for 4 hr. Then, the media was aspirated and 150 μl of DMSO was added for dissolving the formazan crystal by shaking for 10 min. The absorbance at 499 nm was measured to determine the cell viability (Teng et al., 2016).

### 2.8 Glucose consumption and glycogen content assay

The HepG2 cells were seeded in a 96‐well plate at 1 × 10^5^ cells per well and cultured for 24 hr. After modeling, cells were cultured in complete medium with different concentrations of DRE, RAE, and CB (150, 300, 600, 1,200, and 2,500 μg/ml). The insulin resistant (IR) group and the normal control (NC) group were set meanwhile. These five groups were incubated for 24 hr at 37°C in a 5% CO_2_ humidifying atmosphere. The glucose content of culture media was measured by a glucose assay kit according to manufacturer's instruction, and the consumption of glucose was calculated. Glycogen content of cells was determined by anthrone method. The protein content of cells was determined by the total protein assay kit (with standard: BCA method). The glycogen content of cells was presented as the ratio of glycogen/protein (mg/g prot) (Kariya et al., 2019; Wang, He, et al., 2020; Wang, Chang, et al., 2020).

### 2.9 Hexokinase (HK) and Pyruvate Kinase (PK) activity assay

Cell culture and treatment referred to the above methods. The activity of HK was measured by hexokinase test kit and calculated according to the weight of the sample (1 μmol of NADPH which generates in per gram of cells per minute is considered as one enzyme activity unit). Meanwhile, pyruvate kinase test kit was applied to detect PK activity, and PK activity was calculated by the content of intracellular protein (1 mol of PEP conversion to pyruvate in per gram of cells per minute is considered as one enzyme activity unit).

### 2.10 Statistical analysis

Each experiment was performed in triplicate, and the experimental results were expressed as the mean ± standard deviation (*SD*). All data were analyzed by one‐way analysis of variance (ANOVA) followed by Tukey's multiple comparison test with SPSS 22.0 software (IBM Corp.). The Combination Index (CI) was analyzed by CompuSyn software (ComboSyn Inc.).

## 3 RESULTS

### 3.1 Preparation of DRE and quantitative analyses

As shown in Table 1, different solvents had different effects on extraction of dandelion root. DRE‐w had the highest extraction yield which was 24.87 ± 2.18%. The extraction with methanol and ethanol also had high yields which were 15.68 ± 1.46% and 11.19 ± 0.87%, respectively. However, the extraction with other three solvents had a poor yield.

**TABLE 1:** Extraction yield and quantitative analyses of DRE

Extraction solvent	Extraction yield (%)	Chemical composition			
		Polysaccharides /(mg/g)	Total flavonoid /(mg/g)	Total phenolic compounds /(mg/g)	Saponins /(mg/g)
Distilled water	24.87 ± 2.18	63.92 ± 1.82^c^	2.57 ± 0.06^a^	8.93 ± 0.34^c^	0.54 ± 0.05^a^
Methanol	15.68 ± 1.46	6.23 ± 1.54^a^	5.31 ± 0.18^b^	10.21 ± 0.30^d^	0.88 ± 0.03^b^
Ethanol	11.19 ± 0.87	8.26 ± 1.71^b^	10.03 ± 0.24^e^	12.26 ± 0.43^e^	0.8 ± 0.05^b^
Ethyl acetate	1.89 ± 0.09	ND	8.53 ± 0.04^d^	7.17 ± 0.30^b^	0.63 ± 0.03^a^
Chloroform	2.41 ± 0.39	ND	7.44 ± 0.43^c^	8.31 ± 0.24^c^	ND
N‐hexane	1.20 ± 0.12	ND	7.90 ± 0.27^c^	5.73 ± 0.44^a^	ND

Note

ND means not detected. Data are presented as mean ± standard error of the mean (*SEM*). Different letters in the same column indicate significant difference (*p* <.05, *n* = 3).

The content of the biologically active compounds of DRE mainly depends on the solubility of compounds in different solvents. It could be seen from the results that DRE‐w was rich in active compounds; total flavonoid, saponins, polysaccharides, and total phenolic compounds were all detected in relatively higher content, especially the content of polysaccharides in DRE‐w reaching 63.92 ± 1.82 mg/g. A significantly lower content of polysaccharides was detected in the DRE‐m and DRE‐e compared to that in DRE‐w, while contents of total flavonoid, saponins, and total phenolic compounds were significantly richer (*p* <.05). The contents of total flavonoid and total phenolic compounds in DRE‐e were 10.03 ± 0.24 mg/g and 12.26 ± 0.43 mg/g which was significantly higher than that of extract of other solvents (*p* <.05). DRE‐a also had a higher content of total flavonoid; however, polysaccharides were not detected in it. In addition, the polysaccharides and saponins were not detected in either DRE‐c or DRE‐n.

### 3.2 Inhibitory effects on α‐amylase and α‐glucosidase

**FIGURE 1:**
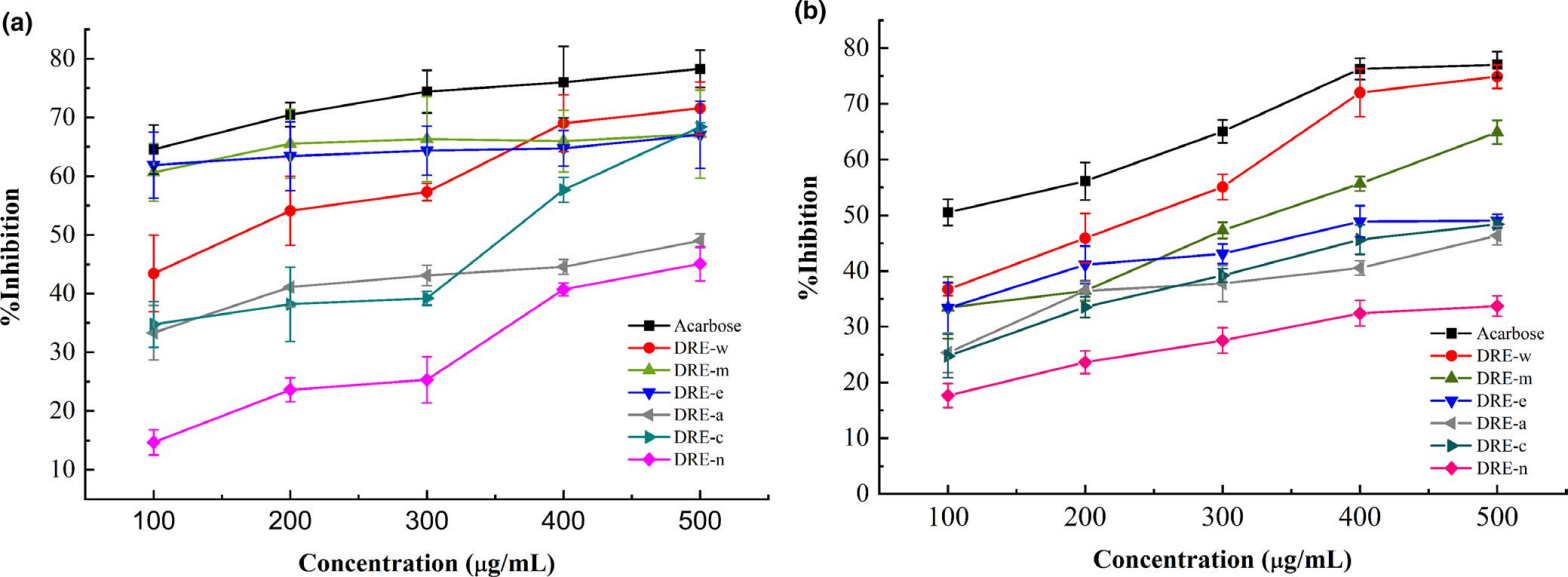
Inhibitory rates of α‐glucosidase (a) and α‐amylase (b) by DREs and Acarbose. Data are presented as mean ± standard error of the mean (*SEM*)

α‐Glucosidase and α‐amylase are common hydrolytic enzymes in the body and can hydrolyze starch into glucose. Figure 1 shows the inhibitory effects of extracts from different solvents on α‐amylase and α‐glucosidase. As the concentration increased, the inhibitory rate basically showed an upward trend in a dose‐dependent manner. When the concentration reached 0.5 mg/ml, the inhibitory rates of acarbose on α‐glucosidase and α‐amylase were 78.28 ± 3.16% and 77.03 ± 2.35%, respectively. Among all the extracts, DRE‐w had the strongest inhibitory effect on enzymes with an inhibition of 71.56 ± 4.49% for α‐glucosidase and 74.9 ± 2.13% for α‐amylase when the concentration was 0.5 mg/ml, which was second only to acarbose. DRE‐m and DRE‐c also had inhibitory effects on enzymes. However, DRE‐n and DRE‐a performed a weak effect of inhibition on α‐glucosidase and α‐amylase.

In addition, the correlation coefficients between each compound in RED‐w and inhibitory rate were determined. As shown in Table 2, the correlation coefficient between polysaccharides and the inhibitory effect on α‐amylase was as high as 0.71. A high correlation coefficient between polysaccharides and the inhibitory effect on α‐glucosidase was also observed, which was 0.36. It indicated that polysaccharides were the functional components that played a major role in the hypoglycemic effect of DRE.

**TABLE 2:** Correlation coefficients between each compound in DRE‐w and inhibitory rate

Correlation coefficients	Chemical composition			
	Polysaccharides	Total flavonoid	Total phenolic compounds	Saponins
α‐amylase	0.36	0.26	0.40	0.17
α‐glucosidase	0.71	0.49	0.48	0.22

In summary, in all extracts tested, DRE‐w contained the highest amount of polysaccharides, which exhibited better inhibition of α‐amylase and α‐glucosidase in vitro and had the highest yield; therefore, DRE‐w was selected as a sample for the following experiments.

### 3.3 Synergistic hypoglycemic effect of DRE‐w and RAE

**FIGURE 2:**
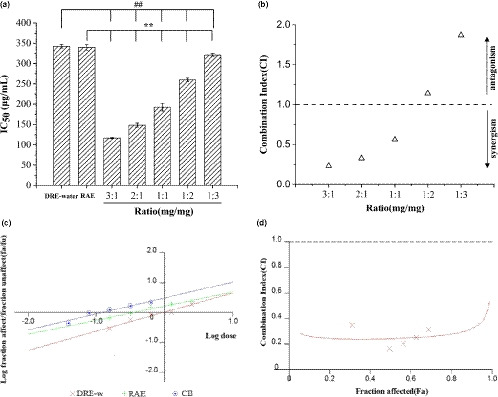
DRE‐w and RAE combination analysis in inhibitory rate of α‐glucosidase: IC_50_ value (a), combination index (b), median‐effect plot (c), and combination index plot of CB (d). Data are presented as mean ± standard error of the mean (*SEM*), ^##^ *p* <.01 compared with the DRE‐w group, **p <.01 compared with the RAE group

DRE‐w and RAE were combined in different ratios, and the α‐glucosidase inhibitory effects of combination were measured. The synergistic effects of DRE‐w and RAE were evaluated according to the IC_50_ values and the CI values. As shown in Figure 2a, when the ratio of DRE‐w and RAE was 3:1, the IC_50_ value was 116.67 ± 1.15 μg/ml, which was 2.94‐fold lower than that of DRE alone and 2.97‐fold lower than that of RAE alone. This indicated that the combination contributed to a lower dose to exert stronger inhibitory effects on α‐glucosidase. The CI values revealed the interaction between the DRE‐w and RAE when they were combined. Figure 2b shows that when DRE‐w and RAE were combined with the ratio of 3:2, 2:1 and 1:1, the CI values were all less than 1 which indicated that the interactions were synergistic between DRE‐w and RAE at this time. The CI value was minimum of 0.231 when the ratio was 3:1. As the proportion of RAE in combination increased, although it could still inhibit the α‐glucosidase activity, antagonism was behaved (CI > 1) between DRE‐w and RAE. Therefore, we selected to combine DRE‐w and RAE in a ratio of 3:1 as the CB for subsequent experiments. According to the median‐effect principle, Figure 2c shows that the dose–effect relationship between CB, DRE‐w, and RAE was further compared; the results were consistent with the above. The results demonstrated in Figure 2d also indicated that DRE‐w and RAE in CB could synergistically inhibit α‐glucosidase activity.

#### 3.3.1 Glucose consumption and intracellular glycogen content in IR‐HepG2 cells

**FIGURE 3:**
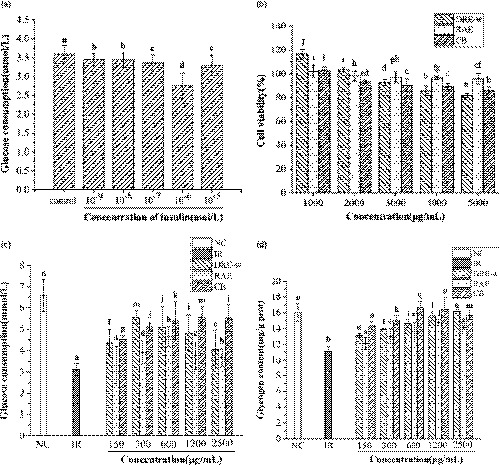
Treatment with DRE‐w, RAE, and CB improved glucose metabolism in IR‐HepG2 cells. Effect of insulin on modeling (a), samples on cell viability (b), glucose consumption (c), and glycogen content (d). Data are presented as mean ± standard error of the mean (*SEM*). Different letters indicate significant difference (*p* <.05, *n* = 3)

In this study, HepG2 cells were used to establish the IR model to evaluate the hypoglycemic activity of DRE‐w, RAE and CB. As shown in Figure 3a, the glucose consumption of the cells decreased after HepG2 cells were treated with different concentrations of insulin. When the concentration of insulin was 10^–6^ mol/L, the glucose consumption of cells was the lowest as 2.756 ± 0.64 mmol/L, reduced by 23.25% compared with control group. Therefore, the concentration of insulin was selected as 10^–6^ mol/L for modeling.

According to the results of MTT assay, the appropriate concentrations of samples were selected for the treatment of IR‐HepG2 cells. Figure 3c shows that the glucose consumption of cells in IR group decreased significantly compared with NC group; however, it increased significantly after the treatment of most of the samples (*p* <.05). When the concentration of samples was 1,200 μg/ml, the glucose consumption of cells in CB group was 5.56 ± 0.51 mmol/L, which was 66.95% higher than that in the model group, 15.08% and 47.92% higher than that in DRE‐w and RAE groups. When insulin resistance occurs, the utilization of glucose by the cells decreases and the synthesis of intracellular glycogen is also inhibited (Priyanka et al., 2017). As could be seen from Figure 3d, the content of intracellular glycogen in IR group decreased significantly (*p* <.05), which was 11.72 ± 0.61 mg/g prot, while the content was 16.04 ± 1.08 mg/g prot in NC group. When the concentration of CB was 600 μg/ml, the content of glycogen in cells was 16.63 mg/g prot, which was higher than that of other groups. The results showed that DRE‐w, RAE, and CB could promote the metabolism of glucose in IR‐HepG2 cells, and CB had a better effect than DRE‐w or RAE treatment alone at the same concentration in general.

### 3.4 Activity of interrelated enzymes in IR‐HepG2 cells

**FIGURE 4:**
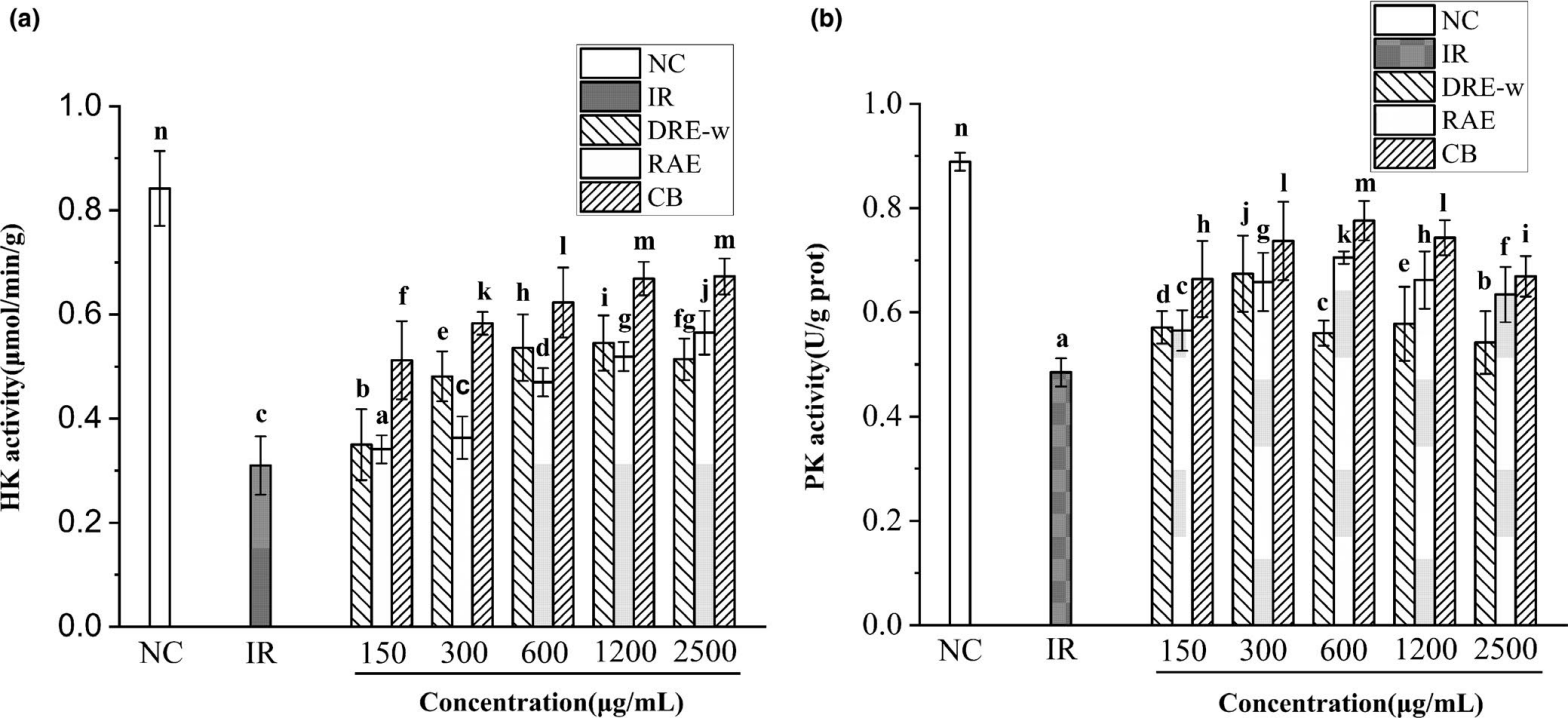
Treatment with DRE‐w, RAE, and CB improved activities of intracellular enzymes in IR‐HepG2 cells. Activity of HK (a) and activity of PK (b). Data are presented as mean ± standard error of the mean (*SEM*). Different letters indicate significant difference (*p* <.05, *n* = 3)

HK and PK are two important enzymes in glucose metabolism. According to Figure 4, compared with NC group, the activity of HK and PK in IR‐HepG2 cells significantly decreased (*p* <.05). After treatment, the activities of HK and PK were all improved. When the concentration of CB was 2,500 μg/ml, the activities of HK and PK were 0.67 ± 0.04 μmol min^−1^ g^−1^ and 0.66 ± 0.04 U/g prot, which were 37.94% and 86.94% higher than that in IR group. In addition, the enzyme activities in CB group were significantly higher than that in DRE‐w and RAE groups. In summary, DRE‐w, RAE, and CB could effectively alleviate the state of insulin resistance in HepG2 cells and this effect was achieved by affecting the intracellular glucose metabolism and the activities of interrelated enzymes. Furthermore, the combination of DRE‐w and RAE achieved a better intervention effect on glucose metabolism in IR‐HepG2 cells.

## 4 DISCUSSION

Dandelion is a traditional medicinal and edible plant, and it has been proved that its roots contained various biologically active compounds. These compounds have different chemical structures which leads them to different solubility in different solvents. In this study, the content of the main active compounds in DRE extracted with different extraction solvents was detected. The results showed that DRE‐w contained a considerable amount of polysaccharide content, and DRE‐e was rich in total flavonoids, saponins, and total phenolic. Polysaccharides and saponins were not detected when the extractants were chloroform or n‐hexane. Significant differences were measured in composition and content of the bioactive compounds in DRE obtained by different extraction solvents, which might cause some differences in biological functions.

The research evaluated the hypoglycemic activity of DRE in vitro according to the inhibitory effect of α‐amylase and α‐glucosidase. α‐Amylase is a hydrolase contained in the human body which can hydrolyze the polysaccharides to produce reducing oligosaccharides and dextrin (Jdir et al., 2015). α‐Glucosidase is a hydrolase distributed on the epithelial mucosa of the small intestine. It can further hydrolyze the oligosaccharides produced by the hydrolysis of polysaccharides into monosaccharides which are easy to absorb. Monosaccharides are converted into blood glucose after being absorbed by small intestinal epithelial cells (Sarikurkcu et al., 2016). Therefore, inhibitors of these two enzymes can be regarded as effective drugs to prevent postprandial hyperglycemia in T2DM patients (Bashary et al., 2020; Gong et al., 2020). In this study, DRE could inhibit the activity of α‐amylase and α‐glucosidase, especially the inhibitory rate of DRE‐w on these two enzymes close to acarbose. It was considered DRE might exert the hypoglycemic effect by inhibiting the activity of α‐amylase and α‐glucosidase and further suppressing the increase of postprandial blood glucose. In addition, studies indicated that the polysaccharides and phenolic compounds in DRE were the main components which exerted inhibition effect of α‐amylase and α‐glucosidase. In previous researches, the hypoglycemic effects of polysaccharides and phenolic compounds have been widely confirmed (Demir et al., 2019; Ganesan & Xu, 2019). DRE‐w was rich in polysaccharides and phenolic compounds; it could more effectively inhibit the activity of α‐amylase and α‐glucosidase; therefore, it was selected for further research.

Currently, researchers have begun to concern about drug combination treatment in diabetes (Johnson et al., 2019; Li & Zhang, 2016; Schlosser, 2019). Studies have shown that the components and contents of the biologically active compounds will change after combination, and the interactions between these compounds may make better therapeutic effects, with fewer side effects due to the decrease in dose of each drug (Cui et al., 2019). As reported previously, RAE has shown a certain hypoglycemic effect and been commonly applied to treat diabetes as traditional Chinese medicine. DRE‐w with RAE was combined to achieve better hypoglycemic effect. The results showed that the combination of DRE‐w and RAE could effectively inhibit the activity of α‐glucosidase. The combination only needed a lower dose to achieve the effect of higher dose of DRE‐w or RAE. Meanwhile, the combined effect was greater than the effect of each drug alone. It was not indicated that the interaction between drugs was synergistic; it might also be additive. The Chou–Talalay method is commonly used for drug combination investigation (Ettari et al., 2020). It can evaluate interaction between drugs through median‐effect equation and combination index (CI). The CI value indicated that DRE‐w and RAE in their combination had shown synergistic hypoglycemic effect. In summary, the combination of DRE‐w and RAE could enhance the hypoglycemic activity.

Insulin resistance is a typical feature of T2DM. When insulin resistance occurs, the glucose metabolism of the liver is disordered, and the uptake and utilization of glucose in liver will be reduced. At the same time, the synthesis of glycogen is inhibited, and the endogenous glycogen is partially decomposed (Fan et al., 2019). In the present research, HepG2 cells were used to establish an insulin resistance model to further study the hypoglycemic effect of DRE‐w, RAE, and their combination in vitro. It was observed that the glucose consumption and intracellular glycogen content of IR‐HepG2 cells had increased after treatments of selected extracts. The combination could relieve the insulin resistance state of HepG2 cells, and it was more effective at the same concentration than RAE or DRE‐w treated alone. Furthermore, results showed that DRE‐w, RAE, and their combination could elevate the activities of HK and PK in the cells. HK and PK are the key rate‐limiting enzymes in the glycolytic pathway. Especially, HK participated in the first step in glycolysis (Yang et al., 2019). It can catalyze the phosphorylation of glucose and convert it to glucose‐6‐phosphate (G‐6‐P). G‐6‐P is also an important reaction substrate in many glycometabolism pathways, including glycogen synthesis. The activities of HK and PK decrease which lead to insufficient glucose utilization by liver cells when insulin resistance occurs. Therefore, we believed that DRE‐w, RAE, and their combination could promote utilization of glucose and synthesis of glycogen by elevating the activity of related enzymes in the cells, thereby alleviating the insulin resistance and achieving the hypoglycemic effect. Similar results were observed in other studies. For example, in the study of baicalein on hypoglycemic effect, baicalein could relieve insulin resistance by regulating the activity of PK and glucokinase (GCK) in IR‐HepG2 cells (Cao, Li, et al., 2019). DRE‐w and its combination with RAE had shown significant hypoglycemic activity, which might be beneficial for the development of related hypoglycemic products. However, the experiments in vivo are still necessary, and the specific mechanism of its hypoglycemic effect needs further studied.

## 5 CONCLUSION

In this study, DRE was prepared with different solvents. When water was used as the extraction solvent, DRE had the best inhibitory activity of α‐amylase and α‐glucosidase. In addition, DRE‐w and RAE had synergistic hypoglycemic effect. DRE‐w and its combination with RAE could relieve the insulin resistance of IR‐HepG2 cells. They could restore the activity of enzymes related to glucose metabolism, increase the consumption of glucose by cells, and promote the synthesis of glycogen. In conclusion, DRE and its combination with RAE could be studied and developed as hypoglycemic functional foods or drugs.

## CONFLICT OF INTEREST

All authors declare that they have no conflicts of interest to this work.
